# Obesity Prevalence in Gaziantep, Turkey

**DOI:** 10.4103/0970-0218.45371

**Published:** 2009-01

**Authors:** Birgul Ozcirpici, Ferhat Coskun, Saime Sahınoz, Servet Ozgur, Ali Ihsan Bozkurt

**Affiliations:** Department of Public Health, Gaziantep University, Faculty of Medicine, Sahinbey, Gaziantep, Turkey; 1 Department of Public Health, Pamukkale University, Faculty of Medicine, Denizli, Turkey

**Keywords:** Gaziantep, obesity frequency, obesity prevalence, turkey

## Abstract

**Background:**

Obesity is associated with reduced quality of life, development of serious chronic conditions such as heart disease and diabetes, increased medical care costs, and premature death. Environmental effects, especially feeding habits may cause hyperinsulinemia and obesity. A Healthy People 2010 objective is to reduce the proportion of adults who are obese to 15%.

**Materials and Methods:**

This cross-sectional study was conducted on 1647 persons in a sample representing Gaziantep, Turkey. Over the selected 329 houses, 310 houses were reached (94.2%) and data about 1315 related persons was collected. The body mass index (BMI) shows the relationship between the weight and the height of people, calculated by the ratio of mass by kg over the square value of height measure. In statistical analyses chi-square, student's *t*-test and logistic regression analysis were used.

**Results:**

The mean BMI increased with time for both sex, whereas decreased for 60+ age group. The fastest increase for both sex was seen while transition from 18 year to 19-29 age groups occurred. Another increase in women was in 30-39 age group; BMI=25.08±4.39 in 19-29 ages whilst BMI=29.02±5.79 in 30-39 ages. The increases in both sex in other age groups were not as much as in this group.

**Conclusion:**

Obesity is not only a problem in the Gaziantep but is also a major health concern in Europe and other regions of the world. As an accepted method against obesity, life-style changes should be put into use from childhood supported in school and family life.

## Introduction

Approximately 1.2 billion people in the world are overweight and at least 300 million of them are obese. According to the World Health Organization (WHO), obesity is one of the 10 most preventable health risks. Obesity, especially abdominal obesity is a very important risk factor of cardiovascular diseases and some types of cancer. It is also conducive to the development of metabolic and rheumatic diseases, diseases of the liver and biliary ducts, as well as respiratory diseases. Obesity has been thought to simply be related to an imbalance between energy intake and expenditure. However, more recent research has suggested that genetic, physiological, and behavioral factors also play a significant role in the etiology of obesity. Discovery of the potential genetic and environmental mechanisms makes the problem not only important for us, but also for our descendances. In other words, obesity is a polygenic, multi-factorial disease occurring by the interaction of genes and environmental factors. According to WHO (1997), the frequency of obesity around the world is 25 percent where 10 percent of those, morbid obeses, have body mass index (BMI) >39. The mean rates of people with normal weight but genetically predisposed to obesity and the rate of people with normal weight is the same, which is 25 percent, in various communities. What makes this epidemia noteworthy is the non-involved population around the world is only 25 percent of it. Recent society which consists of off-springs carrying our ancestors’ fat-prone genes, now comes across with obesity, type-II diabetes, dyslyptemia, hypertension, and arteriosclerosis associated with those factors by the influence of abundant and high-calorie food in the company of inactive living.(1)

The objective of the study was to evaluate the frequency of occurrence of overweight and obesity among the population in the Gaziantep Region, and to determine the relationship between age, gender, marital status, education level, occupation performed, occupational activity and source of maintenance.

## Materials and Methods

A cross-sectional study was applied in a sample representing Gaziantep, Turkey in terms of obesity prevalence.

An optimum sample size which represented rural and urban areas of Gaziantep Province was determined as 1647 (the population of Gaziantep was estimated 1,293,849 as in year 2000). Assuming the mean house populations as 5, the sample space was divided by 5 (1647/5=329). The sample rate of rural over urban sections was estimated by National Statistics Institute (DİE). Over the selected 329 houses, 310 houses were reached (94.2%) and data about 1315 related persons was collected.

The data was collected between June 1, 2003 and June 30, 2003. A face-to-face questionnaire was applied, and height-weight measurement was performed by the academic staff of our department. The weight measurement of the children younger than 2 years was performed by digital scale, whereas the others’ measurement was performed by home-type spring scale with 0.5 kg threshold. The height measurement of the children older than two years and the adults’ were performed while they wore no shoes on smooth surface, heels attached, the hips and shoulders leaned against the wall by using tape measure with 0.1 cm increments. The height measurement of the children younger than 2 years old were performed while standing if possible, otherwise while lying flat on one's back with the help of an adult.

The height versus weight index was used for the children younger than 2 years old. The children having +2 SM higher *z*-scores than the international NCHS/CDC/WHO reference group were attributed as overweighed.

The BMI shows the relationship between the weight and the height of people, calculated by the ratio of mass by kg over the square value of height measure. BMI was calculated for the ages younger- than 2 years old. For the evaluation of ages between 2 and 18, the BMI values developed by Cole *et al*, assuming the BMI=25 as overweighed, and BMI=30 as fat, were used.(2) For the people older than 19 years old, people with BMI between 25 and 29.9 were assumed as overweighed; people with BMI higher than 30 were assumed as fat. In detailed assuming, below are the values:

25.0-29.9 - Overweighed (mildly fat)

30.0-34.9 - First-degree fat

35.0-39.9 - Second-degree fat

40.0+ - Third-degree fat (morbid obese)

The investigation data were evaluated using SPSS 10.0 program. In statistical analyses, chi-square, student's *t*-test, and logistic regression analysis were used.

## Result

The distribution of the attendants’ BMI mean over the age groups and sex were represented in [Table 1]. The mean BMI increased by time for both sex, whereas decreased for 60+ age group. The fastest increase for both sex was seen while transition from 18 year to 19-29 age group occurs. Another increase in women was in 30-39 age group; BMI=25.08±4.39 in 19-29 ages whilst BMI=29.02±5.79 in 30-39 ages. The increases in both sex in other age groups were not as much as in this group.

**Table 1:** Distribution of mean BMI with age and sex

Age groups (Year)	Sex		*P*
		
	Male (Mean BMI±SD)	Female (Mean BMI±SD)	
2-18	17.24±2.91	18.02±3.59	0.007
19-29	23.98±3.60	25.08±4.39	0.041
30-39	25.91±4.41	29.02±5.79	0.001
40-49	26.47±3.47	31.04±6.21	0.0001
50-59	27.75±4.70	31.64±6.53	0.003
60+	25.75±4.43	29.39±4.74	0.001

BMI = Body mass index

The distribution of obesity frequency over age and sex was presented in [Table 2]. The children under the age of 2 years were all overweighed (mildly fat). 11.2% of 2-18 ages were overweighed, whereas 1.8% were fat. 35.9% of the people over the age 19 were overweighed, 26.4% of it were fat at various degrees. Morbid obesity was estimated just in 19+ age group and in women (5.5%). The frequency of overweighness in whole investigation group was 23.7%, and the frequency of obesity at various degrees was 14.4%.

**Table 2:** The distribution of obesity frequency by age and sex in Gaziantep province

	Age groups	Normal		Overweighed		First degree obese		Second degree obese		Morbid obese		Total	
							
		No	%	No	%	No	%	No	%	No	%	No	%
Male	0-1	36	92.3	3	7.7	0	0.0	0	0.0	0	0.0	39	100.0
	2-18	228	90.5	19	7.5	5	2.0	0	0.0	0	0.0	252	100.0
	19+	117	45.2	99	38.2	29	11.2	9	3.5	0	0.0	259	100.0
	Total	381	69.2	121	22.0	34	6.2	9	1.6	0	0.0	550	100.0
Female	0-1	36	90.0	4	10.0	0	0.0	0	0.0	0	0.0	40	100.0
	2-18	261	84.2	44	14.2	5	1.6	0	0.0	0	0.0	310	100.0
	19+	126	30.4	143	34.5	92	22.2	25	6.0	23	5.5	415	100.0
	Total	423	55.3	191	25.0	97	12.7	25	3.3	23	3.0	765	100.0
Total	0-1	72	91.1	7	8.9	0	0.0	0	0.0	0	0.0	79	100.0
	2-18	489	87.0	63	11.2	10	1.8	0	0.0	0	0.0	562	100.0
	19+	243	36.1	242	35.9	121	18.0	34	5.0	23	3.4	674	100.0
	Total	804	61.1	312	23.7	131	10.0	34	2.6	23	1.8	1315	100.0

The variables affecting obesity significantly in 19+ age group were presented in [Table 3]. The frequency of obesity of women in 19+ age group (33.7%) was significantly higher than that of men (14.7%) (*P*<0.0001). The obesity in urban areas (28.9%) was higher than that of the rural areas (20.6%) (*P*=0.024). Smoking and being unmarried reduces obesity (*P*<0.0001). Age was another factor that affects obesity. Working in a job, ethnic origin, and having car affected obesity insignificantly (*P*>0.005) as not foreseen.

**Table 3:** Some variables affecting obesity significantly in 19+ age group

	Not obese		Obese		Total	*P*
				
	No	%	No	%		
Sex						
Male	221	85.3	38	14.7	259	*P*<0.0001
Female	275	66.3	140	33.7	415	
Type of residence						
Rural	162	79.4	42	20.6	204	*P* 0.024
Urban	334	71.1	136	28.9	470	
Marital status						
Married	363	70.6	151	29.4	514	*P*<0.0001
Unmarried	104	92.0	9	8.0	113	
Mate dead	19	54.3	16	45.7	35	
Divorced	9	81.8	2	18.2	11	
Cigarette						
Never smoked	303	67.9	143	32.1	446	*P*<0.0001
Quitted	44	81.5	10	18.5	54	
Still smoking	149	85.6	25	14.4	174	
Age group						
19-29	225	88.9	28	11.1	253	*P*<0.0001
30-39	115	73.2	42	26.8	157	
40-49	61	60.4	40	39.6	101	
50-59	46	51.7	43	48.3	89	
60+	49	66.2	25	33.8	74	

The variables estimated as significant by using logistic regression analysis were presented in [Table 4]. Age (year), chronic disease condition, and sex were the variables that affect obesity. In this analysis, relationship between smoking condition and education status with obesity was insignificant.

**Table 4:** The related factors of obesity by using logistic regression analysis

Independent ariables	*P*	Odds ratio	Confidence interval
Age (year)	<0.0001	1.061	1.050-1.073
Chronic disease status			
No	-	1	1
Yes	0.031	1.562	1.041-2.343
Sex			
Male	-	1	1
Female	<0.0001	3.570	2.308-5.523

## Discussion

It was seen that the mean BMI increased by time for both sex, whereas decreased for 60+ age group. Given that the physical activity and basal metabolism decreased with age after 60 years, the point was the potential obesity problem not the BMI. In addition to decrease in weight after 60 years, it was thought that the people with lower BMI had more chance for long living than those with higher BMI. According to our results, there was a relation between being fat or not with age. The increase in women in all age groups was significantly higher than in men. The BMI of women in 30-39 ages was 29.02±5.79, while it was 25.08±4.39 in 19-29 ages. However, it was thought to be related with pregnancy, the women between 30 and 39 ages should be an important target crowd for preventing obesity. In South-East Anatolian region, it was estimated that the mean BMI of women who had labored in past 5 years and who were not pregnant was 26.5 in Turkey Population and Health Investigation, in 2003.(3) This mean is lower than the women's mean in the study whose age was 19+ (28.18±5.95). If we have look at other countries, according to a study performed in USA in the year 2004, the BMI of the black women of 25+ ages was 27.5±3.37 and BMI of white women was 24.74±5.62 for the same age group; whereas the BMI for women with the ages 18-75 in India was 25.75±4.39.(4,5) However, the age group was not as same as the other studies, the mean BMI value of women in Gaziantep province is higher than the value of the region, the country and, the world. According to Mediterranean values, 25.7 BMI in Greece (17341 people, in 2006, 20-70 ages) and 29.3 BMI in Spain (2001) was estimated.(6,7) There is a resemblance of Gaziantep Province with Spain which is a Mediterranean country in terms of BMI values which could be relied on having similar kitchen culture. The BMI of women in Greece was lower than those in Gaziantep.

In the study, the BMI of men of 19-29 ages and 30-39 ages were 23.98±3.6 and 25.91±4.41, respectively. The BMI of white men over the age 25 in USA was estimated as 22.34±4.12.(4) In India, the BMI for the ages 18-75 estimated was 24.96±3.85.(5) There seems the BMI estimated was higher than values of USA and India. According to Mediterranean data; BMI was 27.3 in Greece (17341 people, in 2006, 20-70 ages), and 28.2 BMI in Spain (2001) was estimated.(6,7) The BMI of men in Gaziantep was lower than the Mediterranean values which could be because of the heavy working conditions, higher fertility in region, and patriarchal families.

According to results of the people at 19+ ages, 35.9% were overweighed and 26.4% were fat at various degrees. In other words, 62.3% of them had weight problems. The same values for women with the same ages were 34.5% and 33.7%; 38.2% and 14.7% for men, respectively. According to Turkey Population and Health Investigation (2003), the overweighed and fat percent among women who had labored in past 5 years and who were not pregnant was 33.5% and 22.3%, respectively while the values estimated for South-East Anatolian region were 58.8% and 23.9%.(3) The frequency of obesity in study group was higher than, both, the region and the country. In the Turkey Obesity Prevalence Study (1999), the frequency of obesity in 20+ population estimated was 22.3%. In the same study, Gaziantep was the second province in terms of obesity frequency as the value 28%.(8) The rural overweighed and obesity frequencies for Erzurum of 20+ women were 28.7% and 14.9%, respectively.(9) The urban overweighed and obesity frequencies for Kayseri of 19+ women were 31.6% and 34.3%, respectively.(10) The residence type affects obesity. The obesity and overweighness seen in Kayseri and Gaziantep, both having publicity about feeding, supports the assumption that the most important factor affecting obesity was feeding. The obesity frequencies for Trabzon of 20+ women were 27.4% and, for men it was 10.7%.(11) The higher physical activity because of the geographical reasons, more share of work for women comparing other regions, and feeding habits should be the possible reasons for lower frequencies than Gaziantep. In Yavuzeli, a county of Gaziantep, the overweighness and obesity frequencies of 40+ ages (2000) were 41.2% and 29.7%, respectively.(12) These frequencies were high despite the indiscriminated sex of 40+ ages. In this study, the obesity frequency was 39.6% in 40-49 ages; and 48.3% in 50-59 ages. In the same region, there has been an increasing obesity frequency for 40+ ages. In USA, the obesity frequency of men has increased from 12.3% (1976-80) to 20% (1988-94) where the change for women was from 16.5% to 24.9% for the same years.(13) In England, the fat and overweighed population had an increase of 15% between the years 1980 and 1992; by the year 1992, 54% of men and 45% of women had involved in overweighness.(14) According to the study commenced by Cardiology Society in Turkey in 1990, the frequency of obesity for men increased from 9 to 19% between the years 1990 and 1999, where it has increased from 19 to 38.8% for women.(15) This increase discloses the obesity problem for the world and the region. This increase in obesity is attributed to increasing dormancy, change in feeding habits, and increase in consumption of delicious and fat-rich food.

The obesity prevalence for some countries according to WHO 2006 Health Statistics Report.(16) The reported frequency for both genders in the table is lower than the mean frequency of the world which may be attributed to geographical, socio-economic, ethnic construct, and genetic differences of those countries. The obesity frequency in Europe is lower than that of our country where it is higher in UAE which may be attributed to feeding habits, genetic factors, socio-economic progress, and factors like life conditions. As one can see, the frequency for women in higher than men throughout the world. This may be because of either physiological factors or of physically lower work-load such as duties involving staying at home and the permanent fat tissue came about in pregnancy. Besides, the obesity frequency in Gaziantep for both genders is higher than the world mean.

Morbid obesity frequency was determined just in 19+ ages and women (5.5% for 19+ women, 3.4% for 19+ general, 1.8% for whole group). The morbid obesity in 19+ women in Kayseri was 2.7%.(10) It was 2% in Yavuzeli county.(12) In USA, in the years 1999-2002, it was 1% for 13-17 ages and 3% for 18-22 ages.(17) In Israel, it was 3.3% for 17+ Women in 2004.(18) Despite the lower rates, the affect of morbid obesity on health and life quality cannot be denied. Besides, in Gaziantep, especially the morbid obesity rates for women are higher than other regions.

11.2% of 2-18 ages were overweighed, 1.8% of them were fat. The same rates for girls were 14.2% and 1.6%; for boys 7.5% and 2%, respectively. In Gemlik region, for 6-12 ages in 2001, it is determined as 16.3% and 2.5% for girls; and 11.6% and 2.1% for boys, respectively.(19) The higher frequency in Gaziantep Region than the other regions for 19+ ages may show the effect of feeding habits on obesity. According to WHO year 2006 Health Statistics Report, the frequencies of overweighness for >5 ages are; 2.2% in Turkey, 5.5% in Romania, 6.2% in South Africa, 1% in Philippines, 2% in China, 5.6% in USA, 2.1% in Pakistan, 5.2% in Mexico.(16) Despite the different age groups studied, one can see that the overweighness frequency was higher than the world mean. This finding is important and dangerous for the province. Obesity has been a social problem that we come across starting early life. For this reason, starting the crusade against obesity in early ages is increasingly ratified.

Taken into account of variables effecting obesity; in women of 19+ ages the frequency (33.7%) was higher than in men (14.7%) (*P*<0.00001), the urban frequency (28.9%) was higher than rural one (20.6%) (*P*=0.024). In USA, the mean BMI is lower in rural and low socio-cultural areas than urban areas.(4) In Peru, in less developed areas determined obesity frequency was found lower.(20) This may be attributed to active life in those areas and, the possible fruit and vegetable centered diet. Also, this makes emphasis on active living yet again.

Smoking lowers the obesity frequency significantly (*P*<0.0001). However, there was no significant difference between those who quitted smoking and still smoking people, but the frequency of obesity in non-smoking group is still high (*P*=0.000000). The findings in USA, Taiwan, and Saudi Arabia also support these data.(4,21,22) There was a reverse proportion with obesity in women and the education status of husbands, daily coffee consumption, and smoking.(23) Including this study, most of the studies’ findings are in agreement with higher frequency of obesity in non-smoking or quitted smoking groups. But, smoking cannot be accepted as a solution for obesity.

To be unmarried lessens the obesity (*P*<0.0001). In USA, the obesity frequency in unmarried people was found lower than the married people.(4) This data may be attributed to higher marriage ages, lesser importance given to physical appearance by the couples when married than in unmarried times, more sedentary life in marriage, regular feeding in marriage, and the hardness of getting rid of the plumps gained in pregnancy.

Another factor affecting obesity was age. The obesity frequency in 19-29 ages was significantly lower than the other age groups. In Trabzon, the highest obesity frequency was in 50-59 ages (34.3%) with the increase until age 60, where it decreased for both genders.(11) The data originating USA show that gaining weight is faster in 20-30 ages while it continues in 30-40 ages.(24) According to the data originating Saudi Arabia in 2002, the obesity prevalence of women in 30+ ages was 49.15% while it was 29.94% for men which had its higher values in 40-49 ages.(22) The forming of obesity is directly related to age, in addition to increasing number of diseases by age. The decrease of metabolic rates and physical activity by mid-ages result the decrease in calorie demand but the continuing of feeding habits may explain the increase in obesity. So, most of the people in 19-29 ages are potential obeses and this group may be a good target intervention group. According to Turkey Population and Health Investigation, in 2003, education status affects BMI but the difference between urban and rural areas is very small.(3)

Age, marital status, count of pregnancy, work and economic conditions are the factors that effect obesity in women.(9) Education, occupation, marital status, income, count of pregnancy, smoking are the factors found in Kayseri that effect obesity.(10) In most of the studies age, gender, genetics, culture, education, socio-economic status, physical activity, ethnic and geographical properties are the factors that are responsible.(20–22,25) As one can see, the factors effecting obesity in the world are similar.

In the study, it was found that the presence of chronic disease increases obesity frequency (*P*=0.031). In the studies in USA, it was found that obesity is a risk factor of cardiovascular diseases, hypertension, high cholesterol, and type-2 diabetes; and it has an increasing involvement in cancer, stroke, respiratory diseases, osteoarthritis, and more of it.(26,27) The risk factor of diabetes and obesity increases with age.(8) In similar studies, it was reported that obesity is a risk factor in hypertension and type-2 diabetes that affects disease prognosis.(11,28) In the study in Istanbul, it was found that obesity is a risk factor of coronary artery disease for both gender, also it was found that the frequency of coronary artery disease in fat women is higher than men.(29) One can think that chronic disease and obesity association may affect each other. Taken with the negative effect of obesity on metabolic system, muscle-skeleton system, digestion system, and heart and vascular structures; obesity may have an importance for all age groups. Taken with its frequency in the world, association with chronic diseases, and the number of risk factors; one can see that obesity is a major health problem that must be cured. The most important step for this cure may be to reduce these risk factors.

As a result of logistic regression analysis, age (year), chronic disease status, and gender were the variables affecting obesity. The risk of obesity increased 1.06 times in every 1-year age increment. Obesity was seen in women 3.5 times more than men which was attributed to metabolic reasons, birth-originating reasons, and increasing sedentary life style because of increasing machine-aided life. Women should be the primary target group of protective programs. Women having chronic disease had 1.5 times more obesity than those not having. But, one can guess that this is not the reason but result, in fact obesity is the reason of chronic diseases.

It is obvious that, obesity is still an important problem of public health in Gaziantep as in the world and so will be in the future. As an accepted method against obesity, life-style changes should be put into use from childhood supported in school and family life. Considering the effect of childhood obesity on type-2 diabetes, one can guess that there will be more number of diabetes and other chronic diseases in the future. According to the studies, one can lose weight more readily in the childhood.(30) Starting from the childhood;

- Educating teachers, students, and kitchen workers about feeding, allowing students to addict to proper food by including nutrient but low-calorie food in school menus.
- With the support of schools and media, education of mothers, especially in obesity-prone families, about preparing proper menus for their children.
- Enabling more active life for children and adults.
- Targeting of especially 20-40 ages adults by obesity preventing programs.
- Pursuing of overweighed and obese people in early-stage and controlling of hypertension and diabetes mellitus.

Should be helpful in crusade against obesity.

## References

[1] Clinical Obesity. Obesity Working Group Publications.

[2] Cole TJ, Belizzi MC, Flegal KM, Dietz WH (2000). Establishing a standart definition for child overweight and obesity worldwide: İnternational survey. BMJ.

[3] (2004). Turkey Population and Health Research, Population Studies Institute of Hacettepe University Turkey 2003. Ankara.

[4] Stephanie A. Robert, Eric N. Reither (2004). A multilevel analysis of race, community disadvantage and body mass index among adults in the US. Soc Sci Med.

[5] Bhansali A, Nagaprasad G, Agarwal A, Dutta P, Bhadada S (2006). Does body mass index predict overweight in native Asian Indians? A study from a North Indian population. Ann Nutr Metab.

[6] Kapantais E, Tzotzas T, Ioannidis I, Mortoglou A, Bakatselos S, Kaklamanou M (2006). First national epidemiological survey on the prevalence of obesity and abdominal fat distribution in Greek adults. Ann Nutr Metab.

[7] Gutierrez-Fisac JL, Lopez E, Banegas JR, Graciani A, Rodríguez-Artalejo F (2004). Prevalence of overweight and obesity in elderly people in Spain. Obes Res.

[8] Satman İ, Yilmaz MT, Şengül AM (1999). Obesity prevalence of Turkey. 22. Turkey Endocrinology and Metabolism Diseases Congress, Abstract book.

[9] Vancelik S, Inandi T, Güraksin A (2003). Prevalence and risk factors of obesity among women 20 years of age and over in a rural area of Eastern Turkey. Turkish J Public Health.

[10] Aykut M, Öztürk Y, Özer A, Aslan A (2002). Obesity of adult women and some factors affecting obesity 8: National Public Health Congress Book. Diyarbakir: October 23-28.

[11] Erem C, Yildiz R, Kavgaci H, Karahan C, Deger O, Can G (2001). Prevelance of diabetes, obesity and hypertension in a Turkish population (Trabzon city). Diabetes Res Clin Pract.

[12] Araz M, Bozkurt Aİ, Ahmet S (2000). Obesity, Hypertension and Diabetes Mellitus Survey of population over 40 year old at Gaziantep City Yavuzeli District. Directions at Endocrinology.

[13] Flegal KM, Carroll MD, Kuzmarzcki RJ, Johnson CL (1998). Overweight and obesity in the United States: Prevalence and trends, 1960-1994. Int J Obes Relat Metab Disord.

[14] Nutrition and Physical Activity Task Force. Department of Health (1995).

[15] Onat A, Sansoy V, Soydan I (2000). Heart health, risk profile and heart disease at Turkish adults: A special study of cardiology.

[16] The World Health Statistics (2006). http://www.who.int/whosis/whostat2006_riskfactors.xls.

[17] Lawson ML, Chen L, Daniels SR, Dolan LM (2005). Prevalence of morbid obesity and metabolic syndrome in US adolescents and very young adults. Ann Epidemiol.

[18] Bar Dayan Y, Elishkevits K, Grotto I, Goldstein L, Goldberg A, Shvarts S (2005). The prevalence of obesity and associated morbidity among 17-year-old Israeli conscripts. Public Health.

[19] Pala K, Aytekin N, Aytekin H (2003). Overweight and obesity prevalence among 6-12 year old children at Gemlik District. Sted.

[20] Jacoby E, Goldstein L, Lopez A, Núñez E, López T (2003). Social class, family anf lifestyle factors associated with owerweight and obesity among adults in Peruvian cities. Prev Med.

[21] Lin YC, Yen LL, Chen SY, Kao MD, Tzeng MS, Huang PC (2003). Prevalence of overweight and obesity and its associated factors: Findings from National Nutrition and Health Survey in Taiwan. Prev Med.

[22] Alsaif MA, Hakim IA, Harris RB (2002). Prevalence and risk factors of obesity and overweight in adult Saudi population. Nutr Res.

[23] Merkus MP, Mathus-Vliegen LM, Broekhoff C, Heijnen AM (1995). Extreme obesity: Sociodemographic, familial and behavioural correlates in The Netherlands. J Epidemiol Community Health.

[24] Rothacker DQ, Blackburn GL (2000). Obesity prevalence by age group and 5-year changes in adults residing in rural wisconsin. J Am Diet Assoc.

[25] Walker AR, Adam F, Walker BF (2001). World pandemic of obesity the situation in Southern African populations. Public Health.

[26] Vandegrift D, Yoked T (2004). Obesity rates, income and suburban sprawl: an analysis of US states. Health Place.

[27] De Onis M, Blossner M (2000). Prevalence and trends of overweight among preschool children in developing countries. Am J Clin Nutr.

[28] Leibson CL, Williamson DF, Melton LJ, Palumbo PJ, Smith SA, Ransom JE (2001). Temporal trends in BMI among adults with diabetes. Diabetes Care.

[29] Sönmez K, Akcakoyun M, Akçay A (2003). Comparison of obesity prevalence, based on body mass index, waist circumference and waist/hip ratio, in patients with coronary artery disease. 73^rd^ EAS Congress book.

[30] Epstein LH, Valoski AM, Kalarchian MA, McCurley J (1995). Do children lose and maintain weight easier than adults: A comparison of child and parent weight changes from six months to ten years. Obes Res.

